# Cranioplasty for Sinking Flap Syndrome in Chronic Subdural Hematoma: A Case Report

**DOI:** 10.7759/cureus.72375

**Published:** 2024-10-25

**Authors:** Wahab Moustafa, Jörg Silbermann, Salah Maksoud, Amr Badary

**Affiliations:** 1 Neurosurgery, Department of Neurotraumatology and Spine, SRH Wald-Klinikum Gera, Gera, DEU; 2 Department of Neurosurgery, Darmstadt Clinical Center, Darmstadt, DEU; 3 Department of Neurotraumatology and Spine, SRH Wald-Klinikum Gera, Gera, DEU

**Keywords:** chronic subdural hematoma, cranioplasty, decompressive craniectomy, sinking flap syndrome, subdural hematoma

## Abstract

Sinking flap syndrome (SFS), a rare complication of decompressive craniectomy, can lead to significant neurological deterioration due to atmospheric pressure changes. Here, we present the case of an 81-year-old male with chronic subdural hematoma who developed SFS post-craniectomy. Initial surgeries involved mini-craniectomy and hemicraniectomy due to acute hematoma complications. The patient experienced progressive neurological deficits, prompting cranioplasty. Following cranioplasty, the patient showed rapid neurological improvement, with resolution of hemiparesis and improved consciousness. Postoperative imaging confirmed stabilization of intracranial dynamics and no new bleeding.

## Introduction

Sinking flap syndrome (SFS), known as paradoxical herniation or the syndrome of the trephined, a rare complication, can arise following decompressive craniectomy [1]. SFS is characterized by neurological symptoms like motor deficits, cognitive disturbances, headaches, and even altered consciousness, often exacerbated in an upright position and alleviated by lying down [2].

While lifesaving, the decompressive craniectomy in conditions such as traumatic brain injury, stroke, or subdural hematoma leaves the brain vulnerable to atmospheric pressure changes, potentially causing the scalp and brain tissue to sink at the craniectomy site [3]. The pathophysiology of SFS involves brain shift, altered CSF dynamics, and local atmospheric pressure on the exposed dura mater, leading to neurological deterioration [4].

The diagnosis of SFS is clinical, supported by imaging studies such as computed tomography (CT) or magnetic resonance imaging (MRI) [5]. These imaging modalities can reveal sinking of the scalp flap, brain shift, and ventriculomegaly. The mainstay of treatment for SFS is cranioplasty, a surgical procedure to repair the cranial defect with autologous bone or synthetic materials [6]. This intervention restores normal intracranial pressure dynamics and protects the brain from atmospheric pressure.

Delays in addressing the condition can lead to persistent neurological deficits and further complications. Cranioplasty not only corrects the mechanical defect but also has profound effects on cerebral hemodynamics and CSF flow, underscoring its role as a critical therapeutic measure [7]. 

This case adds to the literature by highlighting the development of SFS after right-sided hemicraniectomy, with neurological deterioration despite no new bleeding or hydrocephalus and rapid recovery after cranioplasty.

## Case presentation

**Figure 1:**
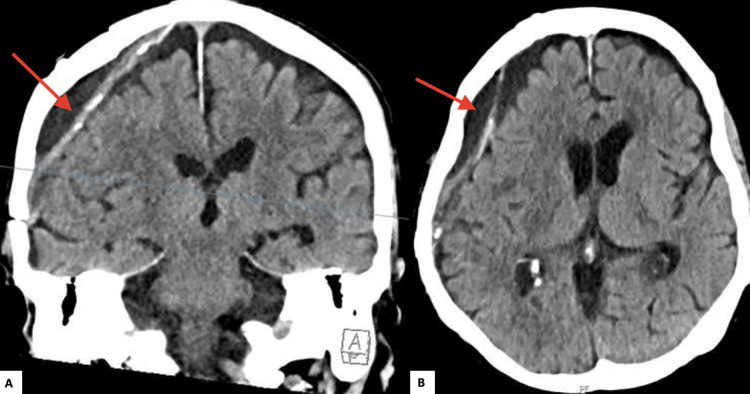
CT scan showing subacute subdural hematoma on the right hemisphere (A: coronal; B: axial). The blue line in the coronal section represents the axial cut section accordingly.

An 81-year-old male presented with a right subacute chronic subdural hematoma midline shift, causing frontal lobe compression and left-sided hemiparesis, which progressed to tetraparesis, left worse than right. A CT scan revealed a 30 mm subdural hematoma on the right hemisphere and a midline shift (Figure 1).

**Figure 2:**
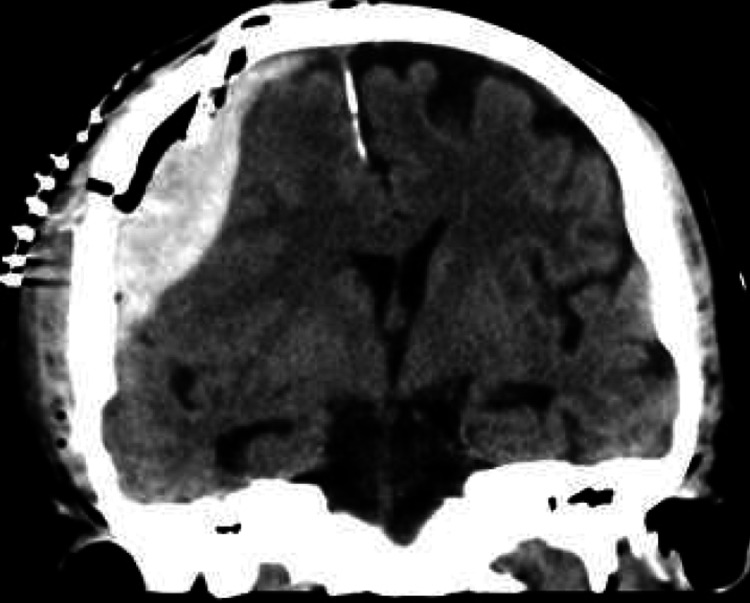
Coronal CT scan showing hyperdense subdural hematoma, partially mixed with air on the right side.

Due to the subacute state of the hemorrhage together with the presence of the epidural membrane, we approached through right-sided mini-craniectomy, the hematoma was washed using room-temperature fluid, and a subdural drain was inserted. The following day, a CT scan showed rebleeding, leading to a second surgery. This scan revealed a 2.7 cm fresh subdural hematoma, bifrontal pneumocephalus, remaining compression of the right lateral ventricle and third ventricle, and a 5 mm midline shift (Figure 2).

During the second surgery, a right-sided hemicraniectomy was performed, and the patient was admitted to the ICU. A subsequent CT scan indicated a significant reduction in bifrontal pneumocephalus but a persistent, narrow, fresh subdural hematoma in the right frontotemporoparietal region. The midline shift remained at 4-5 mm to the left, with continued compression of the right lateral and third ventricles and no signs of herniation.

**Figure 3:**
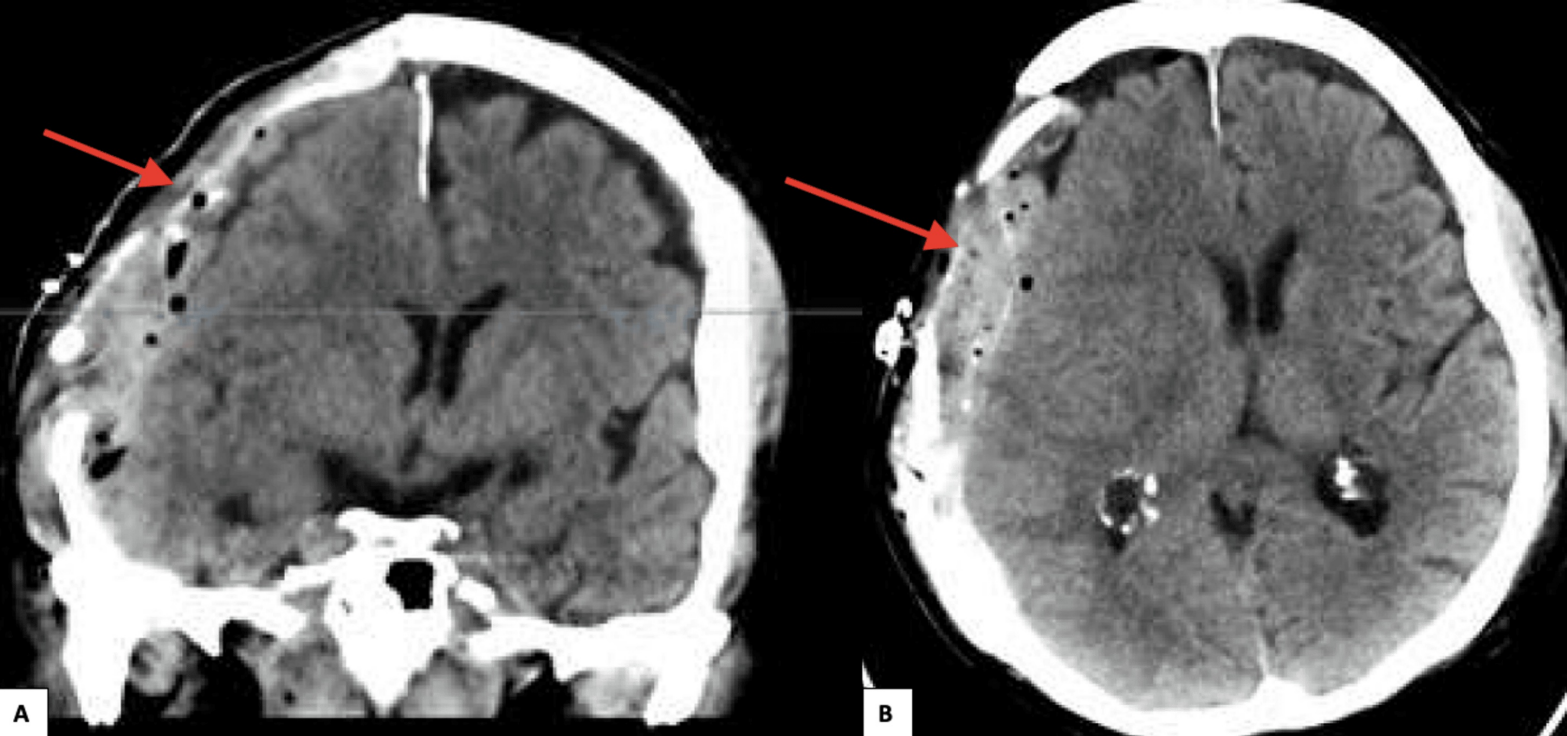
CT scan showing narrow subdural hematoma in the right frontotemporoparietal region (A: coronal; B: axial). The blue line in the coronal section represents the axial cut section accordingly.

Two weeks post-craniectomy, the patient developed additional neurological deficits, including speech impairment and consciousness disturbance. A CT scan revealed a progressive 8 mm midline shift to the left with subfalcine herniation and narrow ventricular spaces, but no new bleeding or hydrocephalus was observed (Figure 3). The presence of subfalcine herniation suggested a potential diagnosis of sinking flap syndrome.

**Figure 4:**
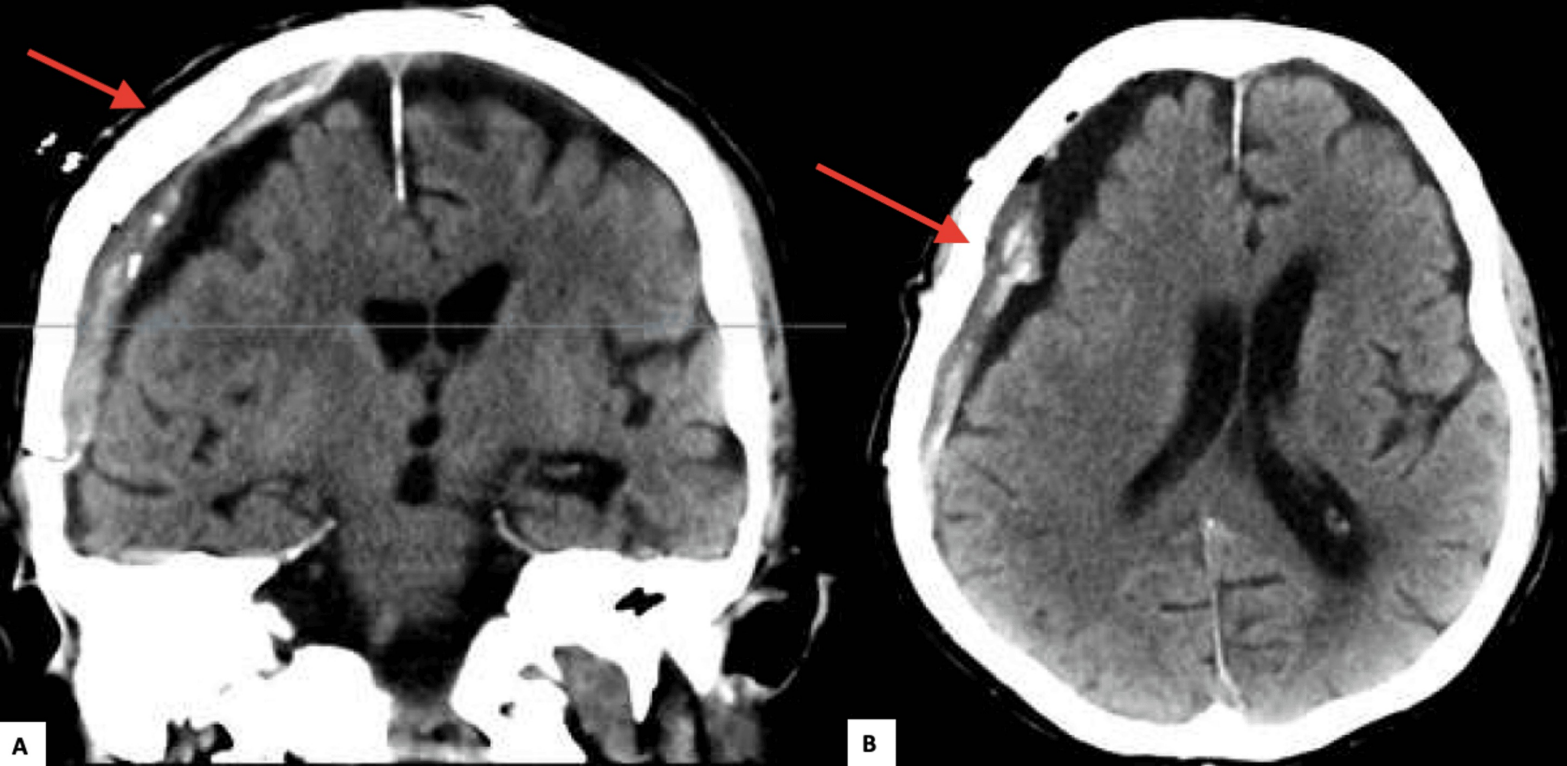
Post-cranioplasty CT scan showing no signs of infarction and no new intracranial bleeding (A: coronal; B: axial). The blue line in the coronal section represents the axial cut section accordingly.

A cranioplasty was performed, and the patient's neurological condition rapidly improved. The paresis resolved, and he was fully awake and responsive. A CT scan performed the day after cranioplasty revealed no infarction, no new intracranial bleeding, and normal widened cerebrospinal fluid spaces with remaining postoperative hygrom (Figure 4), confirming both the improvement following cranioplasty and the diagnosis of sinking flap syndrome.

A month later, a CT scan demonstrated complete regression of postoperative air entrapment and slight narrowing of the right lateral ventricle. The patient continued to recover well.

## Discussion

This case report highlights the critical management of Sinking Flap Syndrome (SFS) in an elderly patient following decompressive craniectomy for a chronic subdural hematoma. SFS results from atmospheric pressure exerting force on the brain through a craniectomy defect. Without the skull as a barrier, atmospheric pressure causes the exposed brain to sink, particularly when the patient is upright, due to reduced intracranial pressure. This pressure imbalance leads to brain shift, impaired cerebral blood flow, and functional deficits. Postural changes, such as sitting or standing, worsen the shift as gravity further decreases ICP, leading to deeper brain collapse [8].

Initially, our patient presented with headaches and left-sided hemiparesis, which progressed to tetraparesis without any speech disturbance. These symptoms are characteristic of chronic subdural hematomas, which typically develop slowly due to the gradual accumulation of blood, resulting in compression of the brain tissue [8].

Radiological evaluation revealed a large right subdural hematoma with a midline shift and ventricle compression. An initial mini-craniectomy was performed, but rebleeding shortly after necessitated a hemicraniectomy. The persistent midline shift and rebleeding highlight the challenges of managing chronic subdural hematomas, especially in elderly patients [9].

A study by Huang et al. (2021) stresses the importance of early surgical evacuation to prevent irreversible brain damage and improve patient outcomes [10]. However, this case also underscores the risk of rebleeding, which has been associated with a poorer prognosis in studies by Shen et al. (2021) [11]. These studies emphasize the critical need for close postoperative monitoring and consideration of additional interventions, such as cranioplasty, to optimize outcomes and mitigate complications.

The decision to perform cranioplasty was pivotal in improving the patient's neurological deficits. Following cranioplasty, there was a marked improvement in the patient's clinical status with resolution of hemiparesis and restoration of consciousness. This outcome is consistent with findings that cranioplasty improves cerebral perfusion and neurological function following decompressive craniectomy [12,13].

The timing of surgical interventions in this case took place two weeks post-craniectomy, as highlighted in the case presentation. Existing literature indicates that timely cranioplasty can significantly improve patient outcomes [4,7,12]. This case emphasizes the crucial importance of prompt decision-making in managing SFS, especially in elderly patients exhibiting suspicious CT findings such as midline shift and narrowed ventricles, to facilitate optimal recovery.

Surgical techniques and management strategies should be refined to optimize outcomes in this vulnerable patient population [14]. As the elderly population continues to grow, addressing these challenges will become increasingly important in enhancing the quality of care and outcomes for patients with decompressive craniectomy.

## Conclusions

This case highlights the development of sinking flap syndrome (SFS) after hemicraniectomy, with neurological deterioration despite the absence of new bleeding or hydrocephalus. Timely cranioplasty led to rapid recovery, demonstrating its critical role in restoring cerebral dynamics and neurological function, particularly in elderly patients. The case emphasizes the need for early diagnosis and intervention in SFS to prevent long-term deficits.

## References

[1] Cassagne M, Claes AS (2022). Sinking skin flap syndrome, a rare complication of craniectomy. J Belg Soc Radiol.

[2] Khan NA, Ullah S, Alkilani W, Zeb H, Tahir H, Suri J (2018). Sinking skin flap syndrome: phenomenon of neurological deterioration after decompressive craniectomy. Case Rep Med.

[3] Vitali M, Marasco S, Romenskaya T (2023). Decompressive craniectomy in severe traumatic brain injury: the intensivist's point of view. Diseases.

[4] Tonini S, Jordanovski D, Williams K (2022). Sinking skin flap syndrome after decompressive hemicraniectomy in a patient with calvarial multiple myeloma who underwent a lumbar puncture: a case report. Cureus.

[5] Sedney CL, Dillen W, Julien T (2015). Clinical spectrum and radiographic features of the syndrome of the trephined. J Neurosci Rural Pract.

[6] Sarti M, Helena T, Daniel P External cranioplasty for the syndrome of the trephined. Interdisciplinary Neurosurgery.

[7] Mah JK, Kass RA (2016). The impact of cranioplasty on cerebral blood flow and its correlation with clinical outcome in patients underwent decompressive craniectomy. Asian J Neurosurg.

[8] Kumar AS, Alugolu R (2014). Chronic subdural hematoma presenting as diplegia-A rare presentation. J Neurosci Rural Pract.

[9] Uno M, Toi H, Hirai S (2017). Chronic subdural hematoma in elderly patients: is this disease benign?. Neurol Med Chir (Tokyo).

[10] Huang Y, Zheng H, Mo M (2021). Effect of different operation time on surgical effect and quality of life in patients with severe hypertensive intracerebral hemorrhage. Am J Transl Res.

[11] Shen J, Shao X, Ge R, Di G, Jiang X (2021). Risk factors for postoperative rebleeding and short-term prognosis of spontaneous cerebellar hemorrhage. Risk Manag Healthc Policy.

[12] Shahid AH, Mohanty M, Singla N, Mittal BR, Gupta SK (2018). The effect of cranioplasty following decompressive craniectomy on cerebral blood perfusion, neurological, and cognitive outcome. J Neurosurg.

[13] Rynkowski CB, Robba C, Loreto M (2021). Effects of cranioplasty after decompressive craniectomy on neurological function and cerebral hemodynamics in traumatic versus nontraumatic brain injury. Acta Neurochir Suppl.

[14] Yadav YR, Parihar V, Namdev H, Bajaj J (2016). Chronic subdural hematoma. Asian J Neurosurg.

